# Emergence of zoonotic *Cryptosporidium*, *Giardia duodenalis* and *Enterocytozoon bieneusi* in wild rodent from Yunnan: first detection of *C. parvum* IIdA24G1 in wild rodents in China

**DOI:** 10.3389/fmicb.2026.1965387

**Published:** 2026-09-17

**Authors:** Rui Duan, Fanfan Shu, Zhiyan Sun, Ping Wang, Hongyi Li, Mengling Deng, Penghao Wei, Xinghe Wang, Fengcai Zou, Jianfa Yang, Luyang Wang

**Affiliations:** 1 College of Veterinary Medicine, Yunnan Agricultural University, Kunming, China; 2 The Yunnan Key Laboratory of Veterinary Etiological Biology, College of Veterinary Medicine, Yunnan Agricultural University, Kunming, Yunnan, China; 3 Yuxi Vocational College of Agriculture, College of Animal Science and Technology, Yuxi, Yunnan, China

**Keywords:** *Cryptosporidium* spp., *Enterocytozoon bieneusi*, *Giardia duodenalis*, wild rodents, Yunnan Province, zoonoses

## Abstract

**Introduction:**

*Cryptosporidium* spp., *Giardia duodenalis*, and *Enterocytozoon bieneusi* are critical zoonotic diarrheal pathogens threatening human and animal health. Wild rodents act as key reservoirs for cross-species pathogen transmission, while systematic epidemiological data of these pathogens in Yunnan rodents remain scarce.

**Methods:**

To elucidate the prevalence and genetic characteristics of *Cryptosporidium*, *G. duodenalis*, and *E. bieneusi* in wild rodents in Yunnan Province, a total of 304 wild rodent samples were collected from seven cities in Yunnan Province, China: Baoshan, Chuxiong, Dali, Diqing, Kunming, Lincang, and Yuxi. Intestinal contents were collected for DNA extraction, and the infection status and species/assemblages/genotypes of *Cryptosporidium* (targeting *SSU* rRNA gene), *G. duodenalis* (targeting *TPI* gene), and *E. bieneusi* (targeting *ITS* gene) in these animals were determined by nested PCR and DNA sequencing.

**Results:**

The results showed that the overall prevalence of *Cryptosporidium* spp., *G. duodenalis*, and *E. bieneusi* were 13.8% (42/304), 18.1% (55/304), and 11.2% (34/304), respectively. The prevalence varied significantly across regions and rodent species. Notably, *Apodemus chevrieri* had a significantly higher risk of *Cryptosporidium* prevalence (OR = 8.70, 95% CI: 2.81–26.94) compared to *Rattus norvegicus*. Eight *Cryptosporidium* species/genotypes: *C. muris, C. meleagridis*, *C. baileyi*, *C. parvum*, rat genotype I-IV, two *G. duodenalis* assemblages (B and G) and five *E. bieneusi* genotypes: three zoonotic genotypes (D, EpbC, Type IV), as well as two known genotype (SDD5, CHY1) were identified. Notably, the zoonotic *C. parvum* IIdA24G1 subtype was first documented in wild rodents in China.

**Discussion:**

Multiple zoonotic genotypes were detected with potential public health risks. This study fills the epidemiological gap of intestinal zoonotic pathogens in Yunnan wild rodents. The newly identified endemic IIdA24G1 subtype and prevalent zoonotic genotypes reveal high spillover risks, providing baseline data for regional pathogen surveillance and public health prevention.

## 1 Introduction

*Cryptosporidium* spp., *Giardia duodenalis* (also known as *Giardia lamblia* or *Giardia intestinalis*), and *Enterocytozoon bieneusi* are the major pathogens responsible for diarrhea diseases in humans and animals worldwide, with *E. bieneusi* being classified as a microsporidial infection, posing a significant threat particularly to immunocompromised individuals (Adam, 2021; Guérin and Striepen, 2020; Nourrisson et al., 2024). These three pathogens are spread chiefly by the fecal-oral route and can infect a variety of animal species, showing particularly high prevalence in wildlife populations. Pathogen-carrying wildlife are highly likely to contaminate water sources. Furthermore, since the pathogens can be transmitted reciprocally among humans, wildlife and livestock, sophisticated transmission chains are ultimately established in their common living environments (Adam, 2021; Nourrisson et al., 2024; Xiao et al., 2004).

Since the discovery of *Cryptosporidium*, over 48 valid species and more than 100 genotypes have been reported, some of which exhibit strong host specificity while others are zoonotic pathogens (Ryan et al., 2021). The majority of human cryptosporidiosis is caused by *Cryptosporidium hominis* and *Cryptosporidium parvum*. In recent years, with the advancement of molecular epidemiological investigations, cases of cryptosporidiosis caused by *Cryptosporidium meleagridis* have gradually increased (Ryan et al., 2014). *Cryptosporidium baileyi* primarily infects avian species; however, waterborne transmission via animal-contaminated water has been suggested, with rare cases reported in humans (Feng and Xiao, 2017; Kopacz et al., 2020). *Cryptosporidium muris* is a gastric Cryptosporidium species that can infect a variety of mammals (Mahdavi et al., 2024). In addition to various rodents, natural infections have also been found in many other mammalian species (Feng et al., 2011). Currently, effective vaccines or drugs against cryptosporidiosis are still lacking. Nitazoxanide, the only drug approved by the U. S. Food and Drug Administration (FDA) for the treatment of human cryptosporidiosis, shows no significant clinical efficacy in immunocompromised or malnourished patients (Amadi et al., 2009). Moreover, the only commercially approved vaccine against cryptosporidiosis is intended solely to protect newborn calves from severe diarrhea, and its efficacy is limited (Timmermans et al., 2024).

*Giardia duodenalis* is classified into eight assemblages or groups (A–H) based on protein and DNA polymorphisms (Klotz et al., 2022). Different assemblages exhibit distinct host distributions. The genotypes infecting humans typically belong to assemblages A and B, which are also found in most mammalian species, indicating broad host specificity and zoonotic transmission (Adam, 2021). In contrast, assemblages C/D, E, F, and G are mainly found in canids, artiodactyls, felids, and rodents, respectively (Zhang et al., 2018). Numerous reports indicate that in children, *G. duodenalis* infection—even in the absence of diarrheal symptoms—may lead to growth retardation or other manifestations of malnutrition (Sackey et al., 2003; Prado et al., 2005; Boeke et al., 2010; Donowitz et al., 2016). For symptomatic giardiasis, there is no specific targeted therapy; the commonly used 5-nitroimidazole drugs, metronidazole and tinidazole, have similar therapeutic efficacy, but cases of treatment failure and drug resistance have been reported (Minetti et al., 2016; Einarsson et al., 2016).

Microsporidia have a broad host range. To date, 10 genera and 17 species have been reported to infect humans, with *Enterocytozoon bieneusi* being the most common pathogen (Zhang et al., 2024). Over 820 genotypes of *E. bieneusi* have been identified in humans and animals, among which 52 are zoonotic genotypes (Koehler et al., 2022). Based on host specificity and zoonotic potential, *E. bieneusi* genotypes are classified into 15 groups (Zhao et al., 2024); groups 1 and 2 contain more zoonotic genotypes, while the remaining groups exhibit stronger host specificity (Koehler et al., 2022). Wild rodents infected with *E. bieneusi* may also carry a greater variety of viruses (Yu et al., 2025), posing a greater threat to humans and other animals. To date, *E. bieneusi* cannot be maintained in in-vitro culture, which limits our understanding of its biology (Nourrisson et al., 2024). The only drug validated to be effective against *E. bieneusi* is fumagillin, a methionine aminopeptidase type 2 inhibitor, whose efficacy has been confirmed in a randomized double-blind placebo-controlled clinical trial (Molina et al., 2002); however, due to its hematological toxicity, its use was discontinued in 2019 (Maillard et al., 2021). Nitazoxanide has also been reported to achieve successful treatment in some cases (Zhou et al., 2023), but for the majority of immunocompromised patients, it still lacks significant efficacy.

Yunnan Province, known for its diverse geography, mild climate, and rich ecosystems, is home to an extraordinary variety of wild rodent species. These rodents not only serve as natural reservoirs for multiple zoonotic pathogens but also frequently act as vectors in the transmission of these pathogens, thereby posing significant threats to public health. *Cryptosporidiosis*, *E. bieneusi* infection, and *G. duodenalis* infection are all parasitic diseases with zoonotic potential, and effective therapeutic drugs are relatively lacking. Therefore, it is particularly important to prevent the occurrence of these diseases, and molecular epidemiological investigations of these three pathogens carry significant public health implications. Wild rodents represent a unique study subject in global surveys due to their remarkable species diversity, worldwide distribution, and habitats that are closely intertwined with human activities. They serve as a link between humans, domestic animals, pets, various pathogens, and wildlife, thereby increasing the risk of rodent-borne disease transmission (Chen et al., 2022). The core concept of One Health is that the health of humans, animals, and our shared environment is inseparable and closely interconnected. To date, no systematic report has been published on the molecular epidemiology of *Cryptosporidium*, *E. bieneusi*, and *G. duodenalis* infections in wild rodents in Yunnan, China. Therefore, this study aims to determine the prevalence and genotype distribution of these three intestinal protozoa in wild rodents in Yunnan Province, adding to the epidemiological data on these pathogens in wild rodents in Yunnan, China, and providing a scientific basis for their prevention and control.

## 2 Materials and methods

### 2.1 Ethics approval and consent to participate

This study involved the capture and euthanasia of wild rodents for sample collection. The procedures followed were reviewed and approved by the Life Science Ethics Committee of Yunnan Agricultural University (IACUC Issue No: APYNAU202503111). The capture, handling, and euthanasia of rodents were carried out in accordance with the *requirements of the Administrative Regulations of Laboratory Animals of China* (2017).

### 2.2 Sample collection

Between March 22, 2025, and January 21, 2026, a total of 304 wild rodents were collected from seven areas adjacent to human settlements or farms in Yunnan Province, China (Baoshan, Chuxiong, Dali, Diqing, Kunming, Lincang, and Yuxi) (Figure 1). These sampling sites were selected to represent different altitudinal gradients (ranging from 511 m to 3,459 m) and climate types (from subtropical to cold-temperate), as well as varying degrees of human-wildlife interface. (Table 1). Rodents were trapped using corn as bait; traps were placed at various rodent-active sites, with approximately 30 traps set each night and retrieved before sunrise. Trapping was conducted for 3–5 consecutive nights at each location, totaling approximately 60–100 trap-nights per site. The collection time and location were recorded for each capture. Within 48 h post-capture, all wild rodents were transported to the laboratory and euthanized by CO_2_ inhalation (Zhao et al., 2018). Sex was recorded, and fresh intestinal contents (500 mg) were immediately obtained from the intestine of each wild rodent, along with 200 mg of muscle tissue. All samples were stored at −20 °C until further use, and DNA extraction was completed within 1 week.

**Figure 1:**
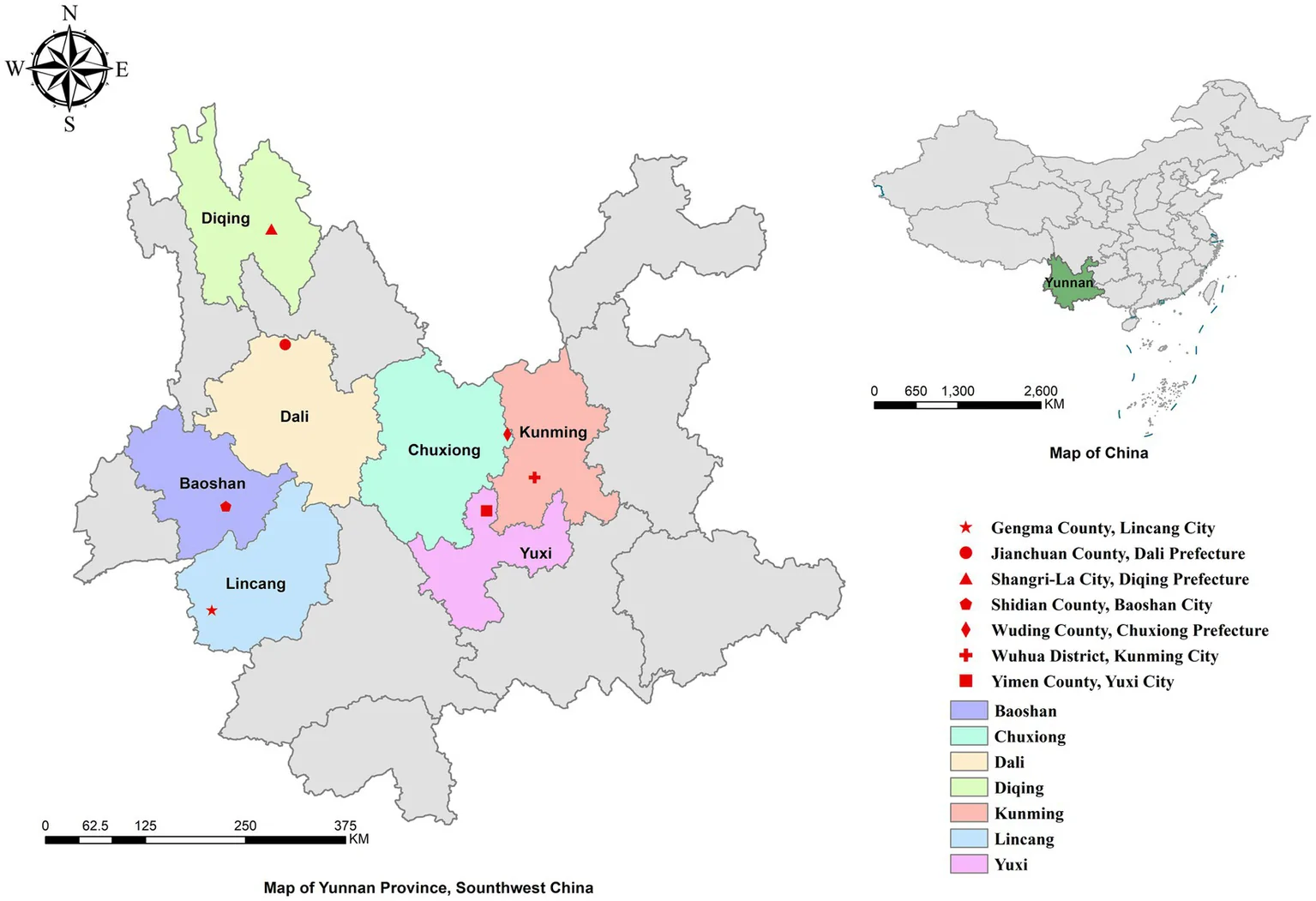
Map of wild rodents sampling sites in Yunnan Province, China. Base map from Tianditu (National Platform for Common Geospatial Information Services), https://www.tianditu.gov.cn/ (Approval No. GS(2024)0650).

**Table 1:** Geographical distribution and sample collection details of wild rodents at seven locations in Yunnan Province, China.

Location	Geographical coordinates	Average elevation (m)	Climate	No. of samples				Total
				*Rattus tanezumi*	*Rattus norvegicus*	*Apodemus chevrieri*	*Rattus nitidus*	
Baoshan	99°14′25.26″E, 24°43′16.94″N	1,460	Subtropical	13				13
Chuxiong	102°24′16.78″E, 25°31′51.46″N	1910	Temperate		34			34
Dali	99°54′23.27″E, 26°32′14.97″N	2,200	Alpine	13	29			42
Diqing	99°45′0.50″E, 27°50′19.15″N	3,459	Cold-temperate		10	15	6	31
Kunming	102°42′31.81″E, 25°2′37.49″N	1887	Mild	1	63			64
Lincang	99°5′0.59″E, 23°33′22.02″N	511	Southern subtropical	56				56
Yuxi	102°10′7.98″E, 24°40′13.23″N	1,585	Mid-subtropical	14	50			64
Total				97	186	15	6	304

### 2.3 Species identification of wild rodents

Species identification of captured rodents was performed using both morphological and molecular biological methods. Morphological identification was carried out primarily following the descriptions in *The Identification Atlas of Medical Vector Organisms at Chinese Border Ports* and *The Classification and Identification Manual of Common Disease Vectors*. Molecular identification was first performed as follows: genomic DNA was extracted from 25 mg of muscle tissue using the Magnetic Beads Animal Tissue Genomic DNA Extraction Kit (Cat. No. DP341; TIANGEN, Beijing, China) according to the manufacturer’s instructions. Subsequently, the mitochondrial cytochrome c oxidase subunit I (COI) was amplified by polymerase chain reaction (PCR) for species identification (Robins et al., 2007).

### 2.4 DNA extraction from intestinal contents

Genomic DNA was then extracted from 200 mg of the intestinal contents samples using an E.Z.N.A.® Stool DNA Kit (OMEGA Bio-Tek, Inc., Norcross, GA, USA) according to the manufacturer’s instructions. The extracted DNA was eluted in 100 μL of elution buffer and stored at −20 °C prior to PCR analysis.

### 2.5 PCR amplification

Nested PCR based on the small ribosomal subunit RNA (*SSU* rRNA) gene was used to detect and identify *Cryptosporidium* species in all DNA samples, with a target product size of approximately 830 bp (Xiao et al., 1999). Nested PCR was also employed for subtype identification of *C. parvum* based on the 60 kDa glycoprotein (*gp60*) gene, with product sizes of approximately 850 bp (Chen et al., 2019). *G. duodenalis* and *E. bieneusi* were detected by nested PCR targeting the *TPI* and *ITS* genes, respectively, to amplify the target fragments, with product sizes of approximately 530 bp and 392 bp (Zhao et al., 2015; Zou et al., 2018) (Table 2). All PCR amplifications were performed with positive controls consisting of *C. andersoni* DNA from cattle for *Cryptosporidium*, *G. duodenalis* assemblage E DNA from pigs for *G. duodenalis*, and *Enterocytozoon bieneusi* EbpC genotype DNA from pigs for *E. bieneusi*, along with a negative control without DNA.

**Table 2:** Primers and PCR conditions used in this study.

Pathogen	Target gene	Primer sequence (5′ → 3′)	Annealing temperature (°C)	Product size (bp)	References
*Cryptosporidium* spp.	*SSU* rRNA	F1: CCCTAATCCTTCGAAACAGGA	55	830	(Robins et al., 2007)
		R1: TTCTAGAGCTAATACATGCG			
		F2: AAGGAGTAAGGAACAACCTCCA	56		
		R2: GGAAGGGTTGTATTTATTAGATAAAG			
*C. parvum*	*gp60*	F1: TTACTCTCCGTTATAGTCTCC	52	850	(Xiao et al., 1999)
		R1: GGAAGGAACGATGTATCTGA			
		F2: TCCGCTGTATTCTCAGCC	52		
		R2: GCAGAGGAACCAGCATC			
*Giardia duodenalis*	*TPI*	F1: AAATTATGCCTGCTCGTCG	58	530	(Chen et al., 2019)
		R1: CAAACCTTTTCCGCAAACC			
		F2: CCCTTCATCGGIGGTAACTT	62		
		R2: GTGGCCACCACTCCCGTGCC			
*Enterocytozoon bieneusi*	*ITS*	F1: GGTCATAGGGATGAAGAG	55	392	(Zhao et al., 2015)
		R1: TTCGAGTTCTTTCGCGCTC			
		F2: GCTCTGAATATCTATGGCT	55		
		R2: ATCGCCGACGGATCCAAGTG			

### 2.6 Sequencing and phylogenetic analysis

Based on the gel electrophoresis results, all secondary positive PCR products were sent to Sangon Biotech (Kunming, China) for bidirectional sequencing on an ABI 3730 sequencer (Applied Biosystems, Foster City, CA, USA). Each raw sequence was assembled using ChromasPro 2.1.5.0^1^. Assembled sequences were compared with GenBank datebase to select appropriate reference sequences. The resulting sequences were then aligned using ClustalX software 2.1.5.0^2^ with *Cryptosporidium*, *G. duodenalis*, and *E. bieneusi* reference sequences obtained from GenBank^3^. A phylogenetic tree was constructed using MEGA 6.0 software^4^ based on the maximum likelihood method. The genetic relationship and reliability of the phylogenetic tree were assessed using the general time-reversible model and by bootstrapping with 1,000 replicates.

### 2.7 Statistical analysis

IBM SPSS Statistics 27.0 (IBM Corp., Armonk, NY, USA) software was used to analyze the effects of region, species, sex and other factors on the infection of wild rodents with *Cryptosporidium*, *G. duodenalis* and *E. bieneusi*. Statistical difference was considered significant at *p* < 0.05. Odds ratios (ORs) with 95% confidence intervals (CIs) were calculated to assess whether the associated factors were risk factors. Prevalence 95% CIs were calculated using the Wilson score method to avoid lower bounds below zero for proportions near 0 or 100%.

Due to the small sample sizes of some rodent species (*Apodemus chevrieri*, *n* = 15; *Rattus nitidus*, *n* = 6) and zero counts in certain subgroups, Fisher’s exact test was used instead of chi-square test when expected cell counts were <5. ORs with 95% CIs were calculated using univariable logistic regression. Multivariable analysis was not performed due to the limited sample size in some strata, which would result in unstable estimates. This limitation is acknowledged in the discussion.

## 3 Results

### 3.1 Wild rodent species

Based on morphological and molecular identification, among the 304 wild rodents collected from seven prefectures and cities in Yunnan Province, there were 97 *Rattus tanezumi*, 186 *Rattus norvegicus*, 15 *Apodemus chevrieri*, and 6 *Rattus nitidus* (Table 1). Analysis of the distribution characteristics of wild rodents across different habitats revealed that *Rattus norvegicus* was mostly distributed in temperate monsoon climate zones, *Rattus tanezumi* was predominantly found in subtropical regions, while *Apodemus chevrieri* and *Rattus nitidus* were distributed in alpine cold regions.

### 3.2 Prevalence of *Cryptosporidium* spp., *Giardia duodenalis*, and *Enterocytozoon bieneusi*

Among the 304 samples collected from seven prefectures and cities, the prevalence of *Cryptosporidium*, *G. duodenalis*, and *E. bieneusi* were 13.82% (42/304), 18.09% (55/304), and 11.18% (34/304), respectively (Table 3). Significant differences were observed in the prevalence of *Cryptosporidium* and *E. bieneusi* across different regions (*χ*^2^ = 16.642, df = 6, *p* = 0.011; and *χ*^2^ = 16.722, df = 4, *p* = 0.002, respectively), while the difference in *G. duodenalis* infection rate was highly significant (*χ*^2^ = 24.018, df = 4, *p* < 0.001). After classifying samples from different regions into three elevation intervals, significant differences were observed in the infection rate of *E. bieneusi* among different elevations (*χ*^2^ = 6.120, df = 2, *p* = 0.047), while no statistically significant differences were found for *Cryptosporidium* (*χ*^2^ = 0.563, *p* = 0.755) and *G. duodenalis* (*χ*^2^ = 0.361, *p* = 0.548). Regarding rodent species, the prevalence of *Cryptosporidium* and *E. bieneusi* differed highly significantly (*χ*^2^ = 18.173, df = 3, *p* < 0.001; and *χ*^2^ = 28.782, df = 2, *p* < 0.001, respectively), whereas the difference in *G. duodenalis* infection rate was not statistically significant (*χ*^2^ = 0.993, *p* = 0.319). For sex, no significant differences were found in the prevalence of the three pathogens (*χ*^2^ = 0.476, *p* = 0.490; *χ*^2^ = 0.347, *p* = 0.556; and *χ*^2^ = 0.126, *p* = 0.723, respectively). Thus, in this study, geographical region significantly influenced the infection of all three pathogens, rodent species significantly affected *Cryptosporidium* and *E. bieneusi* infections, while sex had no impact on the infection status of any of the three pathogens.

**Table 3:** Occurrence and factors associated with *Cryptosporidium* spp. *Giardia duodenalis* and *Enterocytozoon bieneusi* infection in wild rodent in Yunnan Province, China.

Factor	Category	No. tested	*Cryptosporidium* spp.				*G. duodenalis*				*E. bieneusi*			
			No. positive	Prevalence (%) (95% CI)	OR (95%, CI)	*p*-Value	No. positive	Prevalence (%) (95% CI)	OR (95%, CI)	*p*-value	No. positive	Prevalence (%) (95% CI)	OR (95%, CI)	*p*-value
Region	Baoshan	13	4	30.77 (12.68–57.63)	8.89 (1.41–56.25)	<0.05	1	7.69 (1.37–33.31)	Reference	<0.01	2	15.38 (4.33–42.23)	10.00 (0.83–120.16)	<0.05
	Chuxiong	34	2	5.88 (1.63–19.09)	1.25 (0.17–9.37)		18	52.94 (36.34–68.55)	13.50 (1.58–115.70)		–	–	–	
	Dali	42	2	4.76 (1.32–15.79)	Reference		–	–	–		–	–	–	
	Diqing	31	9	29.03 (16.10–46.59)	8.18 (1.62–41.26)		–	–	–		10	32.26 (18.57–49.86)	26.19 (3.16–217.37)	
	Kunming	64	5	7.81 (3.38–17.02)	1.70 (0.31–9.17)		7	10.94 (5.40–20.90)	1.74 (0.17–13.11)		8	12.50 (6.47–22.77)	7.86 (0.95–64.93)	
	Lincang	56	9	16.07 (8.69–27.81)	3.83 (0.78–18.76)		15	26.79 (16.96–39.59)	4.39 (0.35–36.72)		1	1.79 (0.32–9.45)	Reference	
	Yuxi	64	11	17.19 (9.88–28.21)	4.15 (0.87–19.78)		14	21.88 (13.50–33.43)	3.36 (0.40–28.11)		13	20.31 (12.27–31.71)	14.02 (1.77–111.03)	
Average Elevation (m)	>2000	73	11	15.07 (8.63–25.00)	1.23 (0.57–2.70)	>0.05	–	–	–	>0.05	10	13.70 (7.61–23.41)	8.73 (1.08–70.39)	<0.05
	1,000–2000	175	22	12.57 (8.45–18.30)	Reference		40	22.86 (17.26–29.62)	Reference		23	13.14 (8.92–18.95)	8.32 (1.10–63.10)	
	<1,000	56	9	16.07 (8.69–27.80)	1.33 (0.57–3.09)		15	26.79 (16.96–39.59)	1.24 (0.62–2.46)		1	1.79 (0.32–9.45)	Reference	
Rodent species	*Rattus tanezumi*	97	17	17.53 (11.24–26.29)	2.11 (1.02–4.35)	<0.01	22	22.68 (15.48–31.96)	0.74 (0.40–1.35)	>0.05	6	6.19 (2.87–12.83)	Reference	<0.01
	*Rattus norvegicus*	186	17	9.14 (5.78–14.15)	Reference		33	17.74 (12.92–23.87)	Reference		20	10.75 (7.07–16.02)	1.83 (0.71–4.71)	
	*Apodemus chevrieri*	15	7	46.67 (24.81–69.88)	8.70 (2.81–26.94)			–	–		8	53.33 (30.12–75.19)	17.33 (4.69–64.13)	
	*Rattus nitidus*	6	1	16.67 (3.01–56.35)	1.99 (0.22–18.02)			–	–		–	–	–	
Sex	Male	166	25	15.06 (10.41–21.29)	1.26 (0.65–2.45)	>0.05	32	19.28 (14.00–25.95)	1.19 (0.66–2.16)	>0.05	17	10.24 (6.49–15.79)	Reference	>0.05
	Female	138	17	12.32 (7.84–18.84)	Reference		23	16.67 (11.37–23.77)	Reference		17	12.32 (7.84–18.84)	1.23 (0.60–2.51)	
Total		304	42	13.82 (10.39–18.15)	–	–	55	18.09 (14.17–22.81)	–	–	34	11.18 (8.11–15.22)	–	–

No, number; CI, confidence interval; OR, odds ratio.

### 3.3 Distribution of *Cryptosporidium* species and subtypes

Sequence analysis of the *SSU* rRNA gene products from 42 *Cryptosporidium* isolates revealed eight species: *C. muris* (11/42, 26.19%), rat genotype I (9/42, 21.43%), rat genotype II (1/42, 2.38%), rat genotype III (10/42, 23.81%), rat genotype IV (6/42, 14.29%), *C. meleagridis* (3/42, 7.14%), *C. baileyi* (1/42, 2.38%), and *C. parvum* (1/42, 2.38%), with *C. muris* being the predominant species (Table 4). Among these, the *C. parvum* isolate was identified as subtype IIdA24G1 based on *gp60* analysis. In terms of rodent species, only a single *Cryptosporidium* species/genotype was detected in *Apodemus chevrieri* and *Rattus nitidus*, whereas *Rattus norvegicus* and *Rattus tanezumi* exhibited a richer diversity of *Cryptosporidium* species/genotypes due to their broader distribution.

**Table 4:** Species/assemblages/genotypes identification of *Cryptosporidium* spp., *Giardia duodenalis*, and *Enterocytozoon bieneusi* from wild rodent in Yunnan Province, China.

Factor	Category	Species/assemblages/genotypes (*n*)		
		*Cryptosporidium* spp.	*G. duodenalis*	*E. bieneusi*
Region	Baoshan	rat genotype III (3) rat genotype IV (1)	G (1)	D (1) EpbC (1)
	Chuxiong	*C. meleagridis* (1) *C. parvum* (1), IIdA24G1	G (18)	–
	Dali	rat genotype III (1) *C. muris* (1)	–	–
	Diqing	rat genotype I (1) rat genotype IV (1) *C. muris* (7)	–	D (1) SDD5 (9)
	Kunming	rat genotype I (1) rat genotype III (1) rat genotype IV (1) *C. muris* (2)	G (7)	D (6) Type IV (1) CHY1 (1)
	Lincang	rat genotype I (1) rat genotype II (1) rat genotype III (4) rat genotype IV (1) *C. baileyi* (1) *C. muris* (1)	G (15)	D (1)
	Yuxi	rat genotype I (6) rat genotype III (1) rat genotype IV (2) *C. meleagridis* (2)	B (3) G (11)	D (13)
Rodent species	*Rattus tanezumi*	rat genotype I (1) rat genotype II (1) rat genotype III (10) rat genotype IV (2) *C. baileyi* (1) *C. muris* (1) *C. meleagridis* (1)	B (1) G (21)	D (5) EpbC (1)
	*Rattus norvegicus*	rat genotype I (8) rat genotype IV (3) *C. muris* (3) *C. meleagridis* (2) *C. parvum* (1), IIdA24G1	B (2) G (31)	D (17) Type IV (1) CHY1 (1) SDD5 (1)
	*Apodemus chevrieri*	*C. muris* (7)	–	SDD5 (8)
	*Rattus nitidus*	rat genotype IV (1)	–	–
Total		*C. muris* (11), rat genotype I (9), rat genotype II (1), rat genotype III (10), rat genotype IV (6), *C. meleagridis* (3), *C. baileyi* (1), and *C. parvum* (1), IIdA24G1	B (3) G (52)	D (22), SDD5 (9), Type IV (1), CHY1 (1), and EpbC (1)

Sequence analysis showed that certain genetic differences existed among sequences detected from different wild rodent species infected with the same parasite species. However, all 42 isolates shared >98% sequence similarity with the GenBank sequences under accession numbers GU319783, MW090935, PQ362262, MW090930, MK982463, JX294363, JX294365, and MW090928, and were successfully submitted to the NCBI database, obtaining accession numbers PZ437590–PZ437631. For *C. parvum*, the *gp60*-based identification showed 98.99% similarity with OK317684, and the sequence was deposited in NCBI under accession number PZ454254. Based on these results, representative sequences from this study (PZ437631, PZ437608, PZ437612, PZ437607, PZ437605, PZ437604, PZ437599, PZ437591, PZ437593, PZ437590, PZ437625) were used to construct a phylogenetic tree using the maximum-likelihood method together with reference sequences (Figure 2). The different parasite species each clustered with their respective reference sequences, indicating high homology.

**Figure 2:**
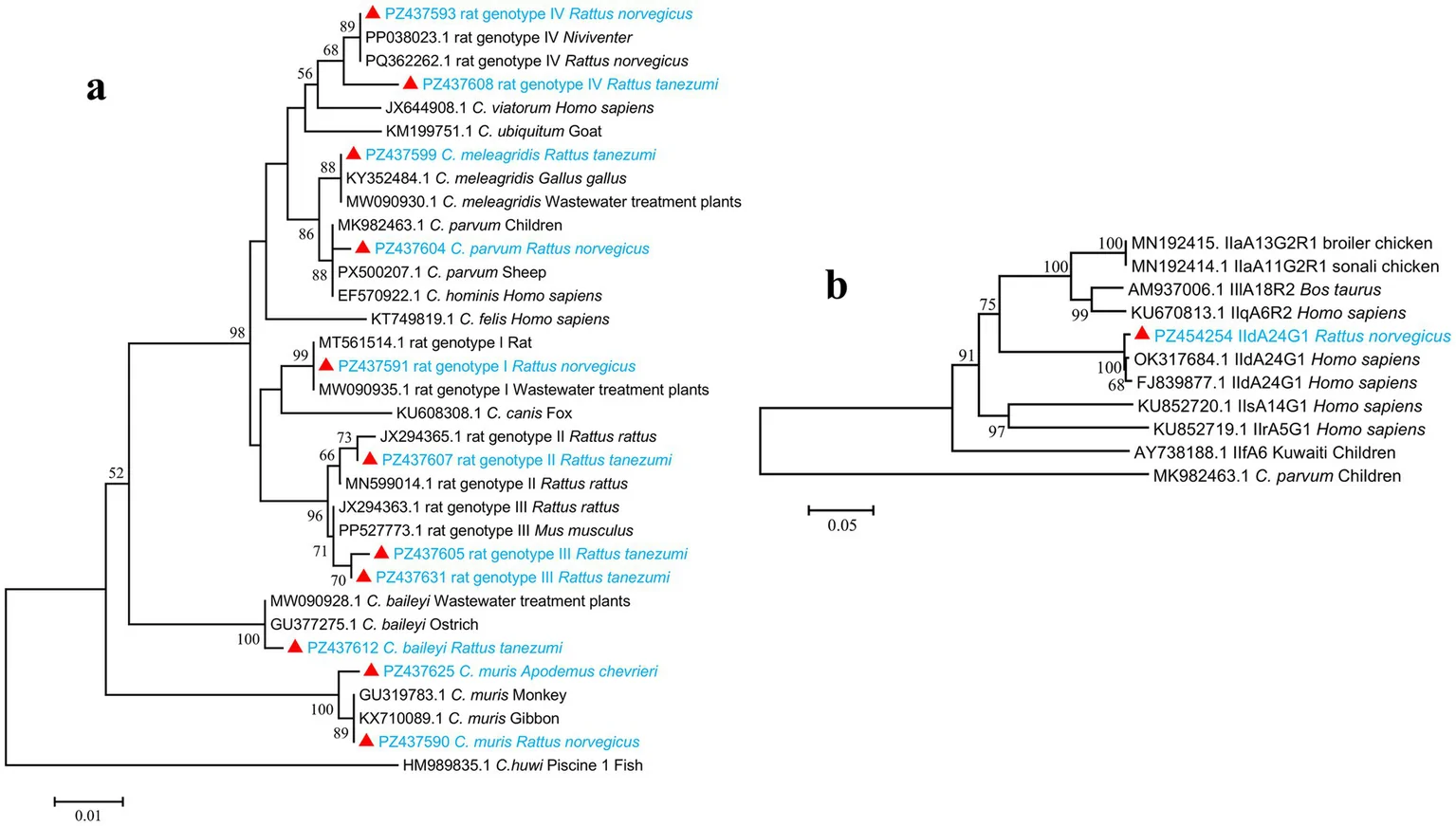
**(a)** Maximum-likelihood phylogenetic tree of *Cryptosporidium* species based on *SSU* rRNA gene sequence. **(b)** Maximum-likelihood phylogenetic tree of *C. parvum* partial genotype based on *gp60* gene sequence. Sequences identified in this study are marked by filled red symbols.

### 3.4 Distribution of *Giardia duodenalis* assemblages

Sequence analysis of the *TPI* gene products from 55 *G. duodenalis* isolates identified Assemblage B (3/55, 5.45%) and Assemblage G (52/55, 94.55%), with Assemblage G being the dominant group, consistent with its characteristic host specificity (Table 4). In this study, Assemblage B was detected only in the Yuxi region, indicating a risk of zoonotic giardiasis transmission from wild rodents in this area.

Sequence analysis showed that the *G. duodenalis* isolates shared >99% sequence similarity with GenBank sequences under accession numbers AY368169 and PP236014. The isolates were submitted to NCBI and assigned accession numbers PZ411443–PZ411497. Based on these results, representative sequences from this study (PZ411443, PZ411444, PZ411447, PZ411457, and PZ411477) exhibited extremely strong homology with reference sequences in the phylogenetic tree (Figure 3), further confirming their assemblage assignment.

**Figure 3:**
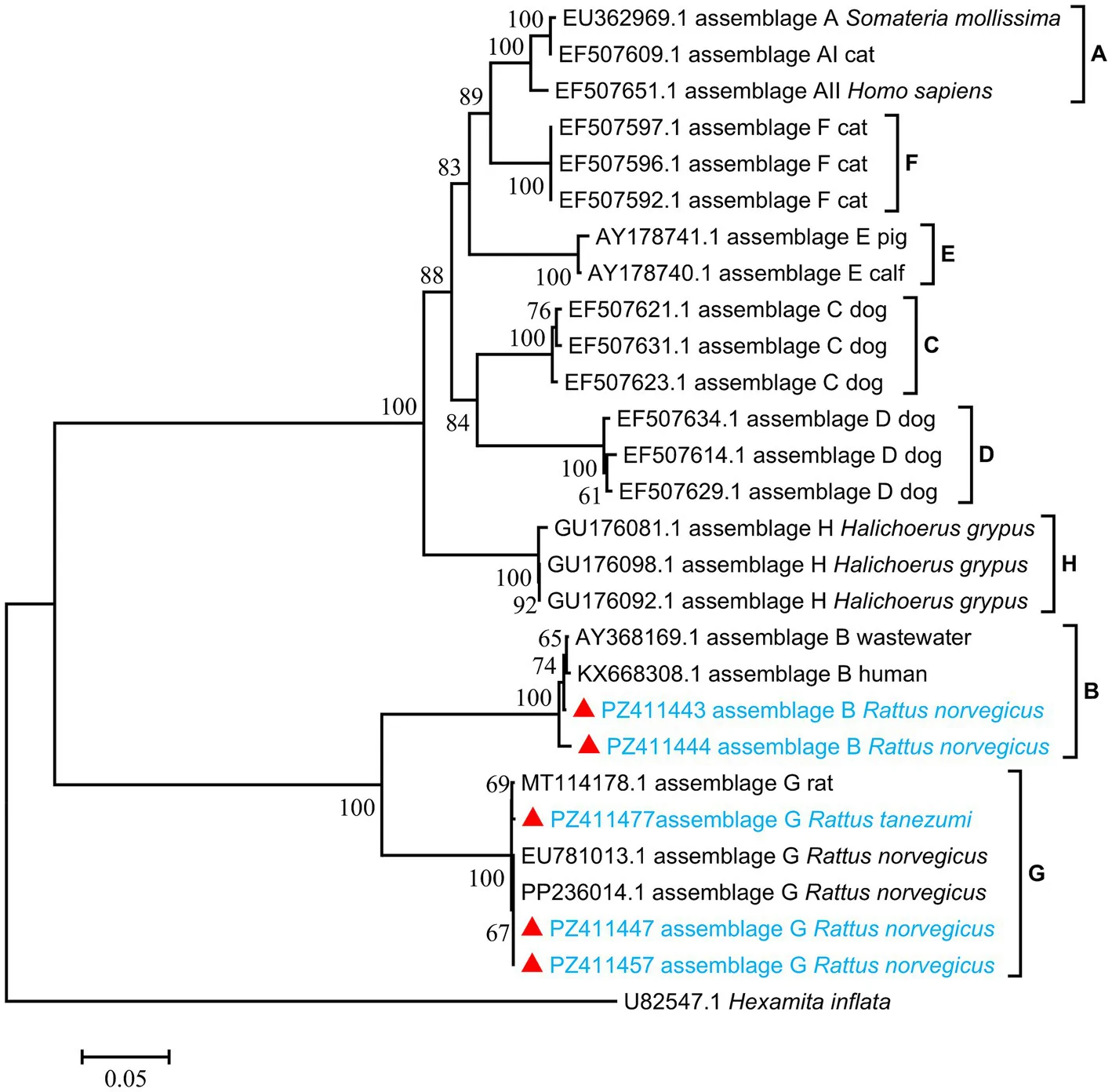
Maximum-likelihood phylogenetic tree of *Giardia duodenalis* assemblages based on *TPI* gene sequences. Sequences identified in this study are marked by filled red symbols.

### 3.5 Distribution of *Enterocytozoon bieneusi* genotypes

Sequence analysis of the *ITS* gene products from 34 *E. bieneusi* isolates identified five genotypes (Table 4): genotype D (22/34, 64.70%), SDD5 (9/34, 26.47%), Type IV (1/34, 2.94%), CHY1 (1/34, 2.94%), and EpbC (1/34, 2.94%). The predominant genotype D belongs to the zoonotic group 1. Except for SDD5 (group 9) and CHY1 (group 10), the other three genotypes belong to group 1, which contains zoonotic genotypes, indicating a substantial zoonotic risk. Genotype D was the dominant genotype in *Rattus norvegicus* and *Rattus tanezumi*, while only genotype SDD5 was detected in *Apodemus chevrieri*, suggesting a relatively specific association between different wild rodent species and *E. bieneusi* genotypes.

Sequence analysis showed that the *E. bieneusi* isolates shared >98% sequence similarity with GenBank sequences under accession numbers PP158568, KJ651436, MN704935, PZ261611, and PP158571. The isolates were submitted to NCBI and assigned accession numbers PZ418477–PZ418510. Based on these results, a phylogenetic tree was constructed together with reference sequences from other groups to further clarify the group assignment of the five genotypes (Figure 4).

**Figure 4:**
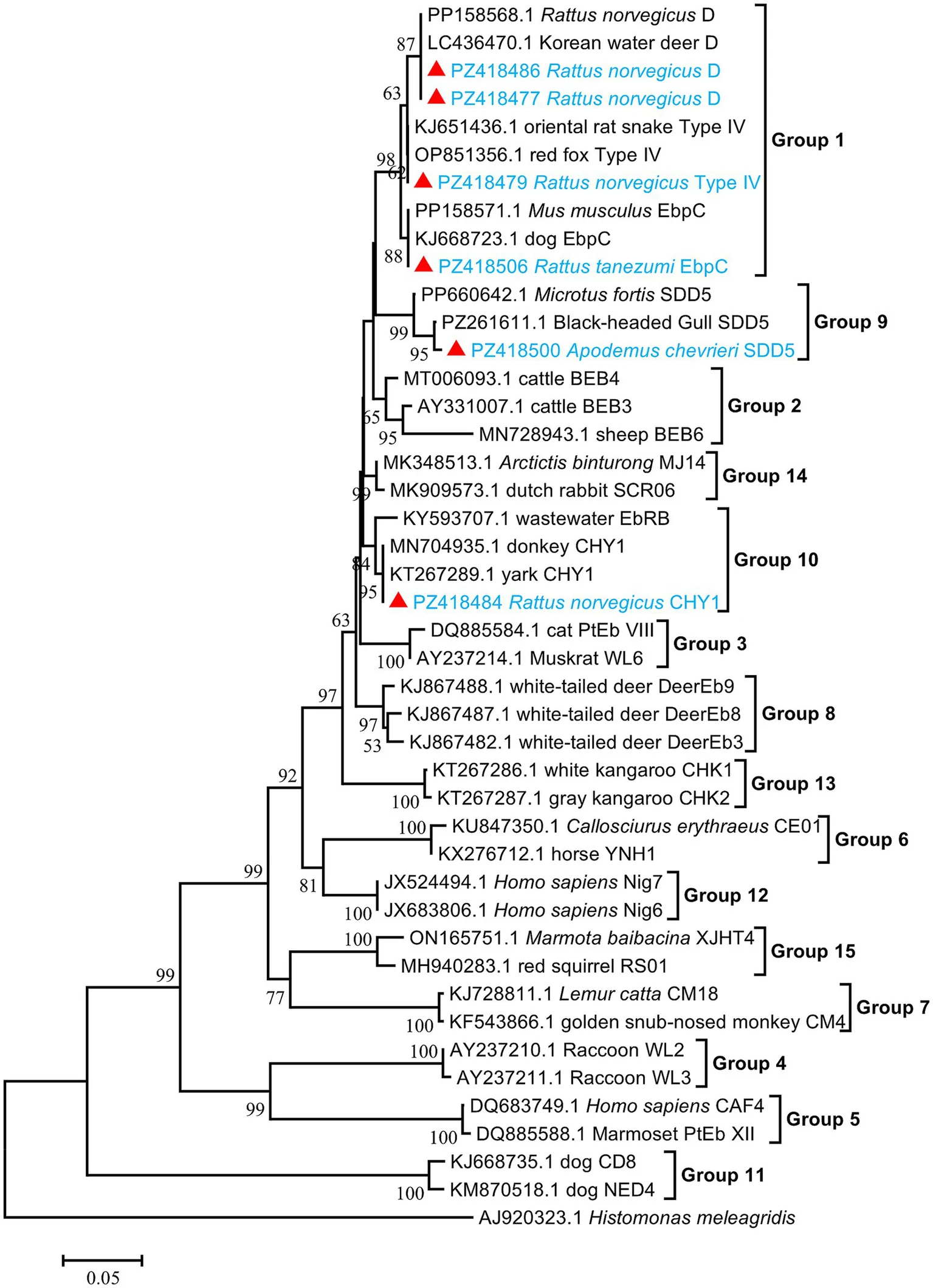
Maximum-likelihood phylogenetic tree of *Enterocytozoon bieneusi* genotypes based on *ITS* gene sequences. Sequences identified in this study are marked by filled red symbols.

## 4 Discussion

The overall infection rate of *Cryptosporidium* in wild rodents in this study was 13.8%, which was higher than those reported in brown rats (*Rattus norvegicus*) in Heilongjiang, China (9.1%) (Zhao et al., 2018), wild rodents in Fujian and Henan provinces, China (8.2%) (Zhao et al., 2015), rodents across China (11.5%) (Lv et al., 2009), and wild rodents in China (3.4%) (Ni et al., 2021). The infection rate was similar to that reported in the same host species in the Canary Islands, Spain (13.86%) (García-Livia et al., 2020), but lower than those in pet rats in Yunnan Province, China (17.2%) (Li et al., 2026) and wild rodents in Malaysia (21.3%) (Tijjani et al., 2020). These differences may be partly explained by geographical and climatic variations. For instance, the high-altitude, cold environment of the eastern Tibetan Plateau (average elevation >4,000 m, annual mean temperature <0 °C) has been shown to prolong oocyst survival in the environment, potentially increasing transmission risks (Zuo et al., 2025). Conversely, lower temperatures may also reduce host activity and contact rates, leading to variable prevalence across regions (Zuo et al., 2025). In addition, host species composition and population density—particularly the dominance of *Rattus norvegicus* and *Rattus tanezumi* in the present study—likely contribute to the observed differences, as different rodent species exhibit varying susceptibilities and roles in pathogen maintenance.

The infection rate of *G. duodenalis* was 18.1%, which was higher than that in wild rodents in Fujian and Henan provinces (6.0%) (Zhao et al., 2015), but lower than that in wild rodents in northeastern Portugal (32.8%) (Lux et al., 2023) and far lower than the prevalence in wild rodents in Germany (73%) (Helmy et al., 2018). The infection rate of *E. bieneusi* was 11.2%, which was higher than that in laboratory rodents in China (4.8%) (Li et al., 2020), but lower than those in wild rodents in Inner Mongolia Autonomous Region and Liaoning Province, China (17.4%) (Zhao et al., 2024) and wild rodents in Xinjiang, Sichuan, and Guangdong provinces, China (12.2%) (Feng et al., 2024), and far lower than that in southwestern Poland (38.9%) (Perec-Matysiak et al., 2015). Notably, previous studies have demonstrated strong host specificity for *G. duodenalis* infections. For example, Zuo et al. (2025) reported that in small wild mammals on the eastern Tibetan Plateau, different host species harbored distinct parasite assemblages. In the present study, the dominant host species—*Rattus norvegicus* and *Rattus tanezumi*—are synanthropic rodents that frequently inhabit human settlements and livestock farms, which may increase their exposure to zoonotic pathogens and facilitate cross-species transmission. This ecological characteristic likely contributes to the observed infection patterns of *G. duodenalis* and *E. bieneusi*.

These differences may be attributed to variations in geographical location, habitat environment, rodent species, sample size, and study design. Moreover, as demonstrated in previous studies, factors such as altitude, climate (especially low temperature favoring environmental persistence of oocysts/cysts), sympatric livestock-wildlife interfaces, and human activities profoundly influence pathogen transmission dynamics (Zuo et al., 2025). In the present study, the infection rate of *E. bieneusi* showed a significant difference across different elevation intervals (*χ*^2^ = 6.120, df = 2, *p* = 0.047), further indicating that elevation-related environmental changes may play a crucial role in shaping the distribution and prevalence of this pathogen in wild rodent populations. The samples collected in this study covered different latitudes and climate types, and the significant differences in prevalence further demonstrated that varying living environments and multiple conditional factors may influence the infection of these three pathogens in wild rodents. Nevertheless, the findings of this study are generally consistent with previous reports from around the world (Li et al., 2026), reinforcing the notion that wild rodents serve as important reservoirs for zoonotic parasites, and that regional ecological contexts must be carefully considered when interpreting prevalence data.

In this study, eight *Cryptosporidium* species were identified. Except for *Cryptosporidium* spp. rat (the rodent-adapted species with strong host specificity), the remaining species—*C. muris* (Wang et al., 2023), *C. baileyi* (Wu et al., 2021), *C. meleagridis* (Penumarthi et al., 2024), and *C. parvum* (Osborn et al., 1977) all pose a risk of cross-species transmission, demonstrating that wild rodents have the potential to transmit these pathogens to humans, livestock, and poultry through contaminated water, although direct transmission remains to be confirmed by epidemiological investigations (Feng and Xiao, 2017). The *C. parvum* identified in wild rodents is a known zoonotic pathogen and one of the two most common *Cryptosporidium* species infecting humans (Nader et al., 2019). Based on the gp60 gene, the genotype of *C. parvum* was identified as IIdA24G1, which is one of the most common genotypes of *C. parvum* (Bujila et al., 2024). This genotype has been found in multiple foodborne outbreaks of *C. parvum* infection in Sweden (Bujila et al., 2024; Gherasim et al., 2012) and has also been reported in a study on *Cryptosporidium* infection in lambs and goat kids in Spain (Quílez et al., 2008); however, it was detected for the first time in wild rodents in the present study. Regarding *G. duodenalis*, this study identified Assemblage B and Assemblage G. Among these, Assemblage B detected in the Yuxi region has a broad host range (Klotz et al., 2022) and was reported as early as 2013 in wild rodents on the Canary Islands, Spain (Fernandez-Alvarez et al., 2014). Therefore, more attention should be paid to the prevention and control of giardiasis in the Yuxi region where this assemblage was detected. For *E. bieneusi*, a total of five genotypes (D, SDD5, Type IV, CHY1, and EpbC) were identified in this study. The presence of these genotypes is not coincidental, as they have all been previously reported in wild rodents infected with *E. bieneusi* across various regions of China (Zhao et al., 2024; Zhao et al., 2020; Gao et al., 2024). Among them, genotypes D, Type IV, and EpbC belong to group 1 (Zhao et al., 2020). Given the widespread infection of wild rodents with zoonotic genotypes of *E. bieneusi*, more precise prevention and control strategies should be developed to curb its transmission and spread.

As wild rodents are the most frequently encountered wild animals in human daily life, the prevalence of zoonotic subtypes of the three pathogens detected in this study raises increasing public health concerns. With human encroachment into peri-urban areas, more infectious *Cryptosporidium*, *G. duodenalis*, and *E. bieneusi* may enter rodent populations (Egan et al., 2024). In particular, pathogen types with broad host ranges are likely to spread from infected wild rodents to humans or livestock over an expanding area. Based on the One Health concept, which recognizes that human health is inseparable from animal health and environmental health, it is imperative to implement hygiene and health promotion measures in urban environments and rural livestock farms to reduce both direct and indirect transmission of these rodent-associated pathogens (Feng et al., 2024).

Several limitations of this study should be acknowledged. Firstly, due to the inherent randomness of wild rodent capture, the sample sizes for certain rodent species were relatively small, which may have limited the statistical power to detect species-specific differences. Secondly, sampling was confined to seven locations within Yunnan Province, and thus the Prevalence reported herein may not be fully representative of the entire province. Furthermore our detection relied solely on PCR amplification of nucleic acids from intestinal contents, which detects DNA/RNA but does not confirm the presence of viable, infective parasites. Finally, the use of single-locus genotyping for *G. duodenalis* (TPI) and *E. bieneusi* (ITS) may have underestimated the true genetic diversity of these pathogens. Future studies incorporating multilocus sequence typing and viability assays are warranted to more comprehensively assess the zoonotic potential and transmission dynamics of these pathogens in wild rodent populations.

## 5 Conclusion

This study reveals the prevalence and genetic diversity of *Cryptosporidium*, *G. duodenalis*, and *E. bieneusi* in wild rodents in Yunnan Province, China. These three pathogens exhibit distinct geographical differences among wild rodent populations. Moreover, the detection of zoonotic subtypes of all three pathogens indicates that wild rodents may serve as potential reservoirs for infection of humans, livestock, and poultry. Therefore, public health education and management should be strengthened to reduce the probability of cryptosporidiosis, giardiasis, and *E. bieneusi* microsporidiosis infection. In future studies, more extensive wild rodent surveillance data and pathogen genetic diversity across broader geographic regions are needed to elucidate the transmission dynamics and public health risks of *Cryptosporidium*, *G. duodenalis*, and *E. bieneusi*.

## Data Availability

The datasets presented in this study can be found in online repositories. The names of the repository/repositories and accession number(s) can be found in the article/Supplementary material.

## Footnotes

1
http://technelysium.com.au/ChromasPro.html/

2
http://clustal.org/

3
http://www.ncbi.nlm.nih.gov

4
http://www.megasoftware.net/
